# Clinico-pathological and biomolecular findings in Italian patients with multiple cutaneous neurofibromas

**DOI:** 10.1186/1897-4287-9-6

**Published:** 2011-08-12

**Authors:** Giovanni Ponti, Lorena Losi, Davide Martorana, Manuela Priola, Elisa Boni, Annamaria Pollio, Tauro Maria Neri, Stefania Seidenari

**Affiliations:** 1 Department of Internal Medicine, Division of Dermatology, University of Modena and Reggio Emilia, Modena, Italy; 2 Section of Pathologic Anatomy, University-Hospital of Modena and Reggio Emilia, Modena, Italy; 3 Molecular Genetics Unit, University-Hospital of Parma, Parma, Italy; 4 Molecular Genetics Unit, Hospital of Reggio Calabria, Reggio Calabria, Italy; 5 Department of Odontostomatological and Maxillofacial Sciences, Oral Medicine Unit, School of Medicine and Surgery, Federico II University of Naples, Naples, Italy

## Abstract

**Background:**

Neurofibroma occurs as isolated or multiple lesions frequently associated with neurofibromatosis type 1 (NF1), a common autosomal dominant disorder affecting 1 in 3500 individuals. It is caused by mutations in the *NF1 *gene, which comprises 60 exons and is located on chromosome 17q11.2. *NF1 *is a fully penetrant gene exhibiting a mutation rate some 10-fold higher compared with most other disease genes. As a consequence, a high number of cases (up to 50%) are sporadic. Mutation detection is complex due to the large size of the *NF1 *gene, the presence of pseudogenes and the great variety of lesions.

**Methods:**

110 patients with at least two neurofibroma lesions recorded in the files of the Pathology Department of the University of Modena during the period 1999-2010, were included in this study. Through interviews and examination of clinical charts, pedigrees were drawn for all patients who were affected by at least two neurofibromas. We attempted to delineate the clinical features of NF1 and the mutational spectrum in the cohort of 11 NF1 families identified. For each proband, the whole coding sequence and all splice sites were studied for mutations, either by the protein truncation test (PTT), or, more frequently, by denaturing high performance liquid chromatography (DHPLC). Two GIST tumors of NF1 patients were tested for somatic NF1 mutations.

**Results:**

NF1 germline mutations were identified in 7 (68%) patients. A novel mutation, c.3457_3460delCTCA in exon 20, was detected in two unrelated patients and was associated with different clinical features. No NF1 somatic mutations were detected in the GIST tumors. A wide phenotypic and genotypic variability was registered, both in the spectrum of skin lesions and visceral neoplasms, even among members of the same family who had different clinical manifestations. A proclivity to multiple tumors arising in the same subject, and a higher tumor burden per family were the most relevant findings observed in patients affected with the NF1 mutation.

**Conclusions:**

We report a novel NF1 mutation and we contribute data for the refinement of the NF1 genotype-phenotype spectrum.

## Background

Neurofibroma is a benign peripheral nerve sheath tumor composed of a variable mixture of Schwann, perineurial-like, and fibroblastic cells that occurs sporadically as well as in conjunction with neurofibromatosis type 1 (NF1) [1-3]. The 50% of patients affected by neurofibromas have no family history of the disease; their NF1 is sporadic and usually results from a new mutation in the germ cell of one of the parents. NF1 (MIM# 162200), also called von Recklinghausen disease or peripheral neurofibromatosis, is one of the most common autosomal dominant disorders, with virtually 100% penetrance by adulthood. The prevalence of NF1 is about 1 in 3,500 live births [4]. Diagnosis is based on the clinical criteria recommended by an NIH Consensus Conference [5], which include multiple café-au-lait spots (CLS), cutaneous or subcutaneous neurofibromas, plexiform neuromas, axillary or inguinal freckling, optic gliomas, and iris Lisch nodules. Although the three characteristic features (CLS, neurofibromas, and Lisch nodules) each occur in over 90% of all NF1 patients by puberty, the number of lesions is extremely variable. Approximately 30-40% of NF1 patients may develop larger and more complex plexiform neurofibromas associated with major nerve trunks [6,7]. These congenital benign tumors often develop in association with the major nerve tracts, where they may involve multiple fascicles and branches of the nerves [8].

NF1 patients are also predisposed to developing dysplastic skeletal lesions, learning difficulties or mental retardation, myeloid leukemias and other malignancies, and may exhibit vascular abnormalities and bone deformities, thus implicating the *NF1 *gene in a wide variety of tissues and disease processes [9,10].

The *NF1 *gene is located at 17q11.2, contains 60 exons spanning approximately 350 kb of genomic DNA, and encodes a 12-kb transcript [11] (GDB: 120231; GenBank: M82814). The gene product, neurofibromin, has an estimated molecular weight of 327 kDa and is widely expressed in many tissues. The mutation rate in the *NF1 *gene is one of the highest reported in any human disorder (approx. 1/10,000 gametes per generation) [12], with approximately 50% of all NF1 patients having no family history of the disease.

The objective of this study was both to evaluate whether a combined clinical and biomolecular approach could be useful for the identification of NF1 Syndrome among a retrospective series of patients with a diagnosis of multiple cutaneous neurofibroma and also to assess the tumor spectrum and potential genotype-phenotype correlation among an Italian cohort of NF1 patients.

## Methods

In total, 331 surgically excised specimens of neurofibromas from 110 patients affected by at least two neurofibromas were retrospectively reviewed by consulting the archives of the Pathology Department of the University of Modena from 1999 through 2010. The inclusion criteria were: a) diagnosis of at least two neurofibromas histologically confirmed; b) tumor tissue available for microscopic analysis (glass slides and/or paraffin blocks); c) medical records with demographic and clinical information. Clinical data (anatomic distribution, gender, age, multiple or isolated lesions, association with NF1, size and duration) were extracted and tabulated. Through interviews and examination of clinical charts, medical pedigrees were drawn for all patients. Detailed family histories were collected for each patient and/or their relatives. Verification of cancer occurrence among family members could be obtained for the majority of patients through clinical charts, pathologic records or death certificates. The qualitative analysis of tumor microscopic features was performed from routine sections stained with hematoxylin-eosin (HeE). Through the reconstruction of the pedigree, we identified the families meeting the consensus NIH clinical NF1 criteria [5].

### Immunohistochemical analysis

For each patient, a representative paraffin-embedded block was sectioned at 4 μm. Immunoperoxidase staining, using diaminobenzidine as chromogen, was run with a Benchmark XT Automatic Staining System (Ventana, Strasbourg, France). The slides were counterstained with hematoxylin. Rabbit polyclonal antibody anti S-100 (Ventana, Strasbourg, France) was used prediluted and MIB-1 mouse monoclonal antibody (anti human Ki-67 antigen, Dako, Glostrup, Denmark) was used at 1:200 dilution.

In addition, mouse monoclonal antibody anti EGFR (clone 31G7, Ventana, Strasbourg, France) was used at a dilution of 1:100 and mouse monoclonal antibody anti p53 (Cell Marque, Rocklin, CA, USA) at a dilution of 1:60.

### Multiplex ligation-dependent probe amplification (MLPA) analysis

Screening for the *NF1 *single and multi-exon deletions was performed using the SALSA P081/082 NF1 V.04 MLPA assay (MRC-Holland, Amsterdam, The Netherlands), following the manufacturer's instructions. This assay consists of two reaction mixtures containing probes for all constitutive *NF1 *exons, with the exception of exons 5, 7, 17, 19a, 45, and 47. An aliquot of ~100 ng of denatured genomic DNA was used in the overnight annealing of the exon-specific probes and subsequent ligation reaction. PCR was carried out with FAM-labeled primers using 10 μl of ligation reaction. Separation and relative quantification of the amplification products were carried out using a Beckman CEQ 2000XL. The peak area for each fragment was measured and normalized by dividing it by the combined area of all peaks in that lane. This normalized peak area was then divided by the average normalized peak area from five normal controls. With this method, the results given are allele copy numbers compared with normal controls, and a ratio of ~1 should be obtained if both alleles are present. A reduction or increase in the peak area values to < 0.7 or > 1.3 was considered an indication of a deletion or a duplication, respectively. DNA samples showing such a reduction or increase in the MLPA peak area values were reanalyzed by MLPA, and only the samples showing consistent results between the two experiments were considered positive for a deletion.

### Germline mutation analysis of *NF1 *gene

Genomic DNA was extracted from the peripheral blood of patients with neurofibromatosis type 1 (NF1) using the QIAamp DNA Blood Mini Kit (Qiagen Inc., Valencia, CA, USA), and stored at -20°C until use. All of the *NF1 *exons were amplified by PCR with intron spanning primers as described by De Luca et al. [13] and analyzed with denaturing high-performance liquid chromatography (DHPLC) as described elsewhere [14]. For each abnormal elution profile, genomic DNA was directly sequenced in both directions using a CEQ Dye-Terminator Cycle Sequencing Kit (Beckman Coulter Inc., Miami, FL, USA) according to the manufacturer's protocol. Mutations were checked using the Mutalyzer program (http://www.LOVD.nl/mutalyzer).

### Testing for somatic NF1 mutations in two GIST tumors

Two patients (no 5 and 6, table 1) developed a GIST tumour. Paraffin-embedded primary tumor specimens of each of their GISTs containing at least 70% tumor cells were selected. For DNA isolation, five 10 μm sections of tumor tissue were used and macrodissected using a safety blade from the normal tissue, scraped off and collected in an Eppendorf tube. Tissue sections were deparaffinated with xilol and washed whith absolute ethanol. Dna was isolated by a silica spin column-based method with the QIAmp FFPE Tissue kit (Qiagne, Hilden, Germany) according to the manufacturer's instructions. Mutation analysis was performed by direct sequencing of 61 constitutive NF1 exons, in both directions, on a 3500 Genetic Analyzer (Applied Biosystems, Foster City, CA) using primers as previously described(Ahiquist T., 2008). Sequencing of coding exons and exon-intron junctions were performed. Data were compared with the mRNA reference sequence and with the chromosome 17 genomic contig reference sequence (NM_000267) [15].

**Table 1:** Clinical and biomolecular features of 11 patients with NF1 syndrome

			Clinical features in the proband					
**Patient N°**	**Gender**, **(age in yrs)**	**Germline mutation**	**NFs (site)**	**Café-au lait spots**	**Freckling**	**Lisch Nodules**	**Other clinical features**	**NFs andTumors in the family** **(age in yrs)**

**Proband 1**	F (16)	Absent	NFs (chest)	> 6 CAL	Present	Present	None	Father: NFs; Liver (69); Duaghter: NF1

**Proband 2**	F (64)	Exon: 20 c.2093_2094delCT p.(Pro698Argfs*3)	NFs (trunk, face, limbs)	> 3 CAL	Axillary and inguinal	N/A	Multiple lipomas, Adrenal adenoma, hepatic angioma, inflammatory polyp of the bowel	Son: Cerebral glioblastoma (30) Son: CAL, NFs

**Proband 3**	M (47)	Exon: 4 c. 479 G > C p.(Arg160Thr)	NFs (trunk, face)	> 6 CAL	Absent	Present	Seminoma, adrenal adenoma	Mother: Breast cancer (59)

**Proband 4**	M (36)	Exon 20 c.3457_3460delCTCA p.(Leu1153Metfs*4)	NFs (abdomen, limbs, face)	> 6 CAL	Axillary	N/A	Plexiform neurofibroma of the forehead, orbito-sphenoid dysplasia, hearing loss, oligophrenia	Mother: NFs;Daughter: NFs Aunt: Larynx (68) Cousin: Pancreas (53)

**Proband 5**	M (50)	Exon 6: large deletion	NFs (Face, Chest, scalp)	> 6 CAL	Axillary and inguinal	N/A	Duodenal GIST, adenocarcinoma of the colon	Mother: NFs; Colon (70); Brother: Colon (59) Uncle: NET (72) Aunt: Lung (52)

**Proband 6**	M (71)	Exon 20 Del c 3457-3460del CTCA p.(Leu1153Metfs*4)	NFs (trunk, face)	> 6 CAL	Axillary	N/A	Duodenal GIST, adenocarcinoma of the rectum	Mother: Renal cell carcinoma (75); Sister: Renal cell carcinoma (49)

**Proband 7**	M (56)	Exon 6 c.750delT p.(Phe250Leufs*31)	NFs (trunk)	> 6 CAL	Axillary and inguinal	Present	Plexiform neurofibroma of the sculp	Brothers:NFs;Lung (70); Son:NFs; Mother:NFs Uncles:NFs Aunt:NFs

**Proband 8**	F (33)	Intron 46, c.8051-70A > T	NFs (trunk and limbs)	> 6 CAL	Axillary	Present	Optic glioma	Aunt: Lung (72)

**Proband 9**	F (38)	Not tested (proband deceased)	NFs (chest, limbs)	> 6 CAL	Axillary	None	Ductal carcinoma in situ, Schwannoma.	Cousin:NFs; Ancle:NFs

**Proband 10**	F(70)	Not tested (proband deceased)	NFs (trunk, limbs)	> 6 CAL	Inguinal	None	None	

**Proband 11**	F (41)	Under investigation	Absent	> 6 CAL	Absent	N/A	Ductal carcinoma (41)	Daughter: > 6 CAL(4) Grandfather: NFs, ADK colon (65) Uncle: bown sarcoma

**NFs: **Neurofibromas; **NET: **neuroendocrine tumor **CAL: **Café-au lait spots; **N/A: **information not available.

### Real time PCR

DNA copy-number changes identified by MLPA were confirmed using an ABI 7700 Sequence Detection System (Applied Biosystems) and the DNA-binding dye SYBR Green (Applied Biosystems). To account for possible variations related to DNA input amounts or the presence of PCR inhibitors, the reference gene *MEFV *exon 5 was simultaneously quantified in a separate tube for each patient sample. SYBR Green amplification mixtures (25 μl) contained SYBR Green master mix, 150 nmol/l of each forward and reverse primer, and 60 ng of template DNA. The PCR cycling conditions were as follows: 2 min at 50°C, 2 min at 95°C, 40 cycles of 95°C for 15 s and 60°C for 30 s, and a final step at 72°C for 30 s. After PCR amplification, a melting curve was generated for every PCR product to check the specificity of the PCR (absence of primer dimers or other nonspecific amplification products). Each assay included a no-template control, 60 ng of a normal control DNA used as a calibrator, and approximately 10 ng of test DNA (in triplicate). Each sample was combined with two non-deleted negative controls (in triplicate). The threshold cycle (Ct) values of SDS software V.2.3 (Applied Biosystems) were exported to Excel (Microsoft Corp., Seattle, WA, USA) for further analysis. The ΔΔCt calculation for the relative quantification of target was as follows:

where χ is the unknown NF1 sample and *y *is the calibrator.

Results for each sample were expressed in N-fold changes in χ*NF1 *gene copies, and normalized to MEFV relative to the copy number of the *NF1 *gene in the calibrator according to the following equation [16]:

A reduction in the peak area values to < 0.7 was considered an indication of a deletion.

## Results

**Figure 1:**
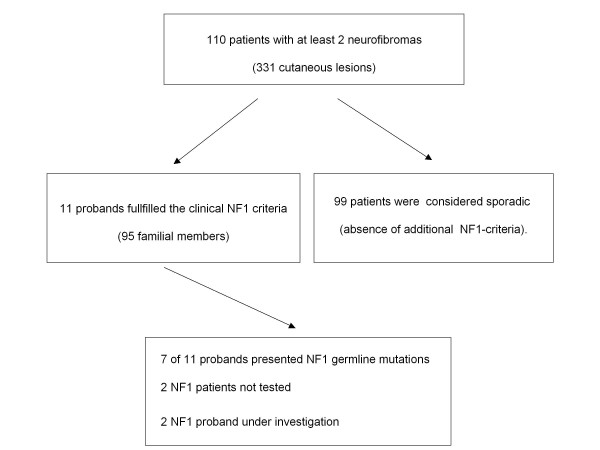
**NF1 cases selected and the subsequent break-up in subcategories**.

**Figure 2:**
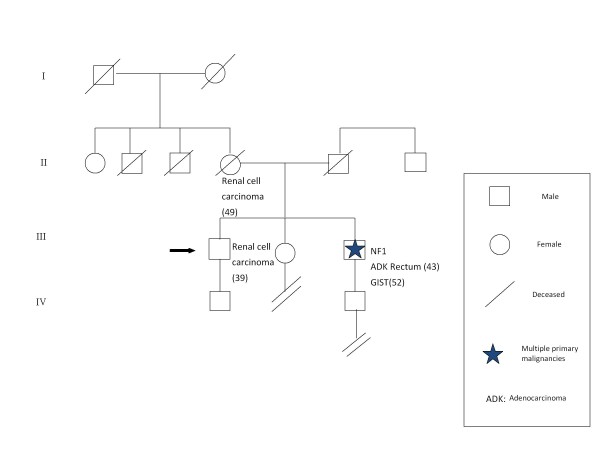
**Family pedigree of NF1 proband with GIST and neurofibromas cosegregating *NF1 *exon 20 germline mutation (c.3457_3460 del CTCA)**.

**Figure 3:**
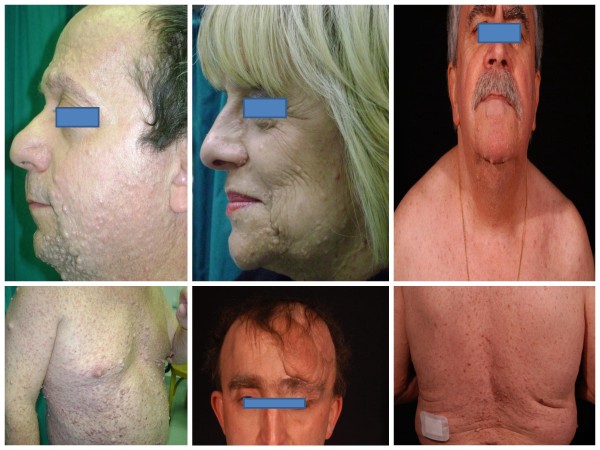
**Clinical features of NF1 probands**.

Clinical characterization and family history of all patients (*n *110 patients) were obtained. Familial NF1 was diagnosed in 11 of 110 patients who had at least two neurofibromas (Figure 1; Table 1). All of the remaining 99 patients were categorized with sporadic cutaneous neurofibromas. The NF1 patients with a positive family history of neurofibromas and other skin lesions typical of NF1 fulfilled the criteria for a clinical diagnosis of NF1; this familial NF1 cohort included 95 patients (37 males and 58 females) who showed all skin lesions characteristic of NF1, including café-au lait macules, neurofibromas and peripheral nerve sheath tumors (Figure 2, 3).

Most sporadic (64%) and most NF1-associated (53%) neurofibromas were located in the supraclavicular brachial plexus and the remainder were in an infraclavicular location. In patients affected by neurofibromas associated with NF1, skin neurofibromas were often multiple, synchronous, or metachronous, and they were located preferentially in the brachial plexus (53%) followed by the upper extremity (29%) and lower extremity (18%). The average age at onset of the first skin neurofibromas was 45.5 years (range, 14-86 years). Cutaneous neurofibromas are found in the majority of NF1 individuals, usually develop in the late teens or early twenties but occasionally emerge in early childhood [17].

**Figure 4:**
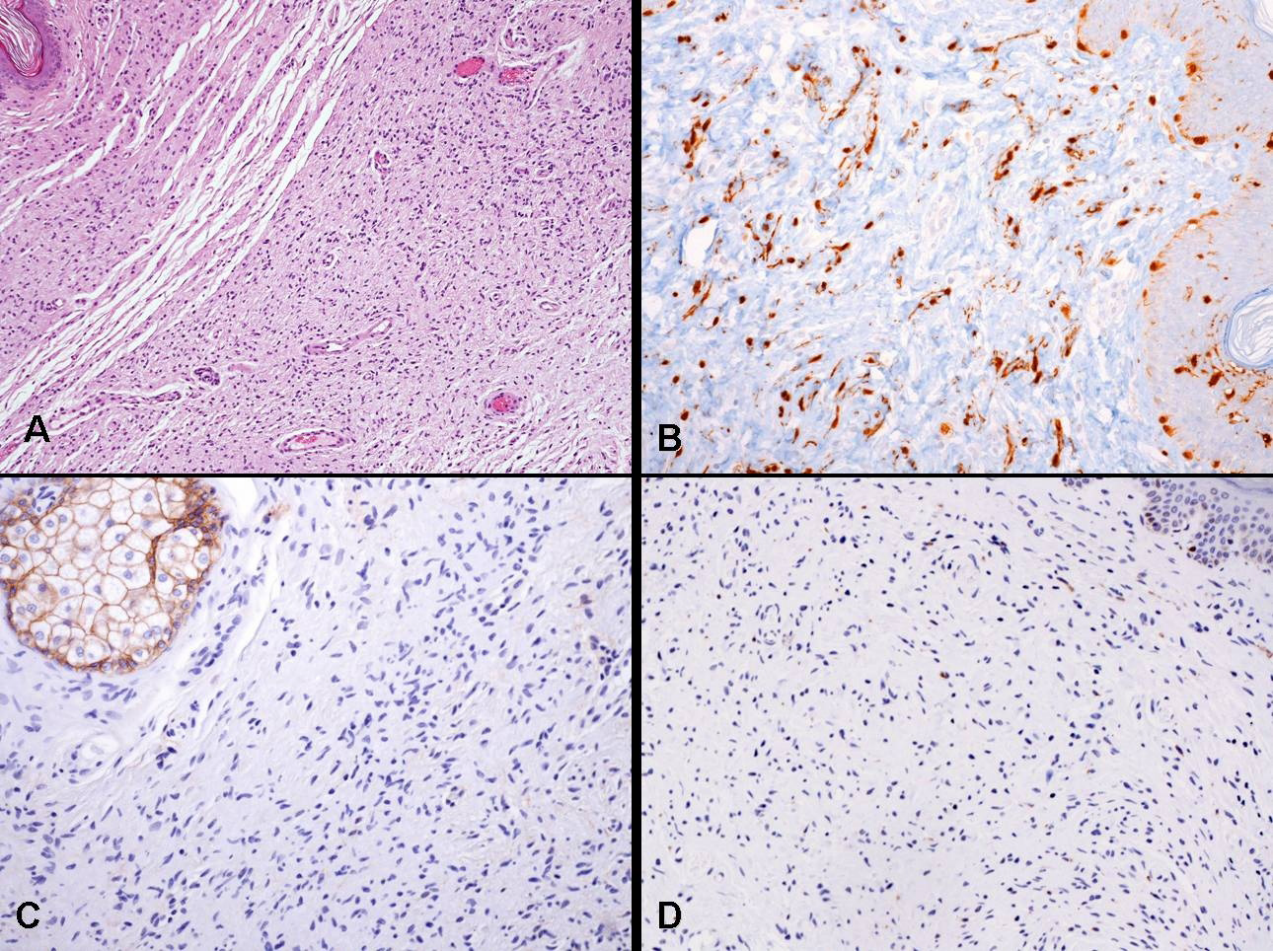
**A) Histological features of neurofibroma (HE) (10 ×); B) Immunohistochemistry showing S-100 immunoreactivity (20 ×) C) Immunohistochemistry showing absence of expression of EGFR (20 ×); D) Immunohistochemistry showing absence of expression of p53 (20 ×)**.

Clinically, the neurofibromas were soft, slightly elevated, and rarely exceeded 2 cm in maximum dimension. The neurofibromas in our sample developed in later life, and only one patient confirmed that it was present at birth. For all we examined all skin neurofibromas resected surgically. Microscopically, neurofibromas were formed by a combined proliferation of all elements of a peripheral nerve: axons, Schwann's cells, fibroblasts. In the majority of cases, the predominant element was Schwann's cell, immunoreactive for S-100 protein. Mitosis was exceptional. The immunohistochemical analysis of the examined neurofibromas showed absence of expression of EGFR and p53 (Figure 4).

In the NF1 cohort patients, the average age at onset of the first visceral malignancy was 52.3 years. The tumor spectrum in the NF1 probands included adenomatous polyps, two colonic adenocarcinomas, two gastrointestinal stromal tumors (GISTs), and one breast tumor. Among first-degree affected relatives, adenomatous polyps, cerebral glioblastoma, colon cancers, breast cancer and pancreatic cancer were detected (Table 1).

Among the 11 NF1 probands, two GISTs and two colorectal carcinomas were found (Patients 5 and 6 in Table 1). Histologically, the GISTs were predominantly epithelioid consisting of mildly to moderately atypical medium-sized polygonal cells. Both the GIST patients had multiple cutaneous neurofibromas and more than six pigmented macules ranging up to 1,5 cm in diameter. One of them underwent left hemicolectomy for T3N0M0 well differentiated adenocarcinoma of the rectum at 43 years of age. Subsequently, a small tumor was resected at the serosal side of the duodenum. Pathologic examination showed GIST with very few mitoses (less than 5 per 50 high power fields). He had a mother and brother who were similarly affected by kidney cancer (Figure 2). In NF1 families, a wide phenotypic and genotypic variability was evident, both in the spectrum of skin lesions (neurofibromas, café-au-lait macules) and in the type of visceral neoplasms. A great phenotypic variability was revealed, even among members of the same family who had different clinical manifestations.

The results of the biomolecular analysis of 11 consenting Italian patients with histologically confirmed multiple neurofibromas, reported seven germline NF1 mutations (Table 1). The identified constitutional *NF1 *gene mutations ranged from single base pair substitutions to gross deletions and the microlesions appeared to be uniformly distributed across the gene. Two identical mutations (c.3457_3460delCTCA) in exon 20 were associated with different clinical features in two unrelated NF1 families but to common proclivity to multiple tumors arising in the same subject and a higher tumor burden per family (Patients 4 and 6 in Table 1).

No somatic NF1 mutations were detected in GIST neoplasms of patients 5 and 6 (Table 1).

## Discussion

A combined clinicopathological and biomolecular approach could be useful for the identification of NF1 Syndrome among patients with a diagnosis of multiple cutaneous neurofibromas, starting with the evaluation of retrospective pathological records. The majority of the neurofibromas in this series were sporadic neurofibromas not associated with NF1. These results clearly indicate that most NF lesions develop as sporadic forms, confirming the discriminatory role of an accurate family history that should be viewed as the crucial step for a proper diagnosis of neurofibromatosis type 1. In particular, the family history can be very helpful in making the diagnosis in young children with only CAL spots, but the role of the family history is limited as almost half of all patients with NF1 are the first to be affected in their families. Surgically treated benign and malignant peripheral neural sheath tumors have been described in a large series by Kim et al. [18] which showed that neurofibromas were more often solitary lesions and not associated with NF1. Our cut-off of at least two neurofibroma lesions as inclusion criteria represented a preliminary screening of patients affected by a solitary lesion not usually associated with NF1. It is known that the other 50% of patients affected by neurofibromas have no family history of the disease; their NF1 is sporadic and perhaps results from a new mutation in the germ cell of one of the parents. Molecular biologists have observed that about 90% of new NF1 mutations are paternally derived [19] and some studies have investigated the hypothesis that older paternal age may increase the risk for a new germinal NF1 mutation [20,21]. However, the causes of sporadic neurofibromas not associated with NF1 remain in part unclear. The trauma has been proposed to trigger the formation of a neurofibroma [22-24]. Yla-Outinen et al. [25] have demonstrated that *NF1 *gene expression is up-regulated during wound healing in humans who do not have NF1.

In our NF1 patient cohort, a great phenotypic variability was revealed both among unrelated patients and even among members of the same family who had different clinical manifestations. However, besides the typical cutaneous lesions, the tumor spectrum of our NF1 cohort included several gastrointestinal neoplasms. Of interest, in two of the 11 NF1 unrelated probands described in this report, we find the diagnosis of GIST. Documented gastrointestinal manifestations of NF1 include neurofibromas, most commonly in the jejunum, ganglioneuromatosis leading to disordered gut motility, gangliocytic paragangliomas, periampullary duodenal carcinoid tumors and GISTs [26,27]. The clinical association between GIST and type 1 neurofibromatosis has been well characterized [28,29]. However, little is known about the molecular basis underlying GIST development in neurofibromatosis as well as in other syndromic settings, and whether there is a difference in the molecular pathogenesis of sporadic GISTs and GISTs associated with multi-neoplastic disease.

Our research into the second, that is somatic, NF1 mutation in the GISTs of both probands carrying the NF1 germline mutations started with the hypothesis that the second somatic hit would appear to be an independent event in each NF1-associated tumor. We did not observe any somatic NF1 mutations in the two GIST tumors studied, but our analysis would not have identified possibly relevant deep intronic NF1 mutations or NF1 rearrangements. The somatic mutation spectrum of the *NF1 *gene might be a crucial factor explaining the variable clinical expression observed in NF1 patients. Evaluation of additional NF1 patients with GIST is necessary for better clarification of GIST pathogenesis in the NF1 setting.

The identified germline *NF1 *gene mutations reflect the wide genotypic variability ranging from single base pair substitution to gross deletions and the microlesions appeared to be uniformly distributed across the gene. Until now, the genotype-phenotype relationship has not been comprehensively studied in patients harboring NF1 mutations or large *NF1 *gene deletions.

Among our NF1 families, two identical mutations (c.3457_3460delCTCA) in exon 20 (Patients 4 and 6 in Table 1) did not cause similar clinical features in two unrelated NF1 patients. One of them had multiple neurofibromas, developmental abnormalities, multiple CLS, axillary freckling, duodenal GIST and rectal adenocarcinoma, while the other had developmental delay, multiple CLS and plexiform neurofibromas but had no GIST. These observations suggest that disruption of the *NF1 *gene itself may not be enough to account for the clinical variability and for the diverse NF1 phenotypes. Some investigators have attempted to explain the variable clinical expression seen in patients with identical germline *NF1 *mutations. In the case of co-existing GIST and other GI tumors, which mostly arise in the upper GI tract, second NF1 somatic mutations might play a significant role. Alternatively, inherited or epigenetic modifiers, such as ethnicity or somatic aberrations in other genes, may determine distinct clinical phenotypes. As approximately half of the clinically diagnosed NF1 population can be classified at the molecular level as having neurofibromatosis type 1 (i.e. proven presence of a germline mutation in *NF1*), there are most likely other genomic regions that are also responsible for the disease and it has been hypothesized that mutations in genes other than *NF1 *may be necessary for the development of the full phenotype. Some studies have evaluated the role of *EGFR *and *p53 *genes in the pathogenesis of malignant transformations of NF1 into MPNST: *EGFR *and *p53 *were expressed in Schwann cells in 26% of MPNST [30,31]. Our immunohistochemical evaluation of neurofibromas does not show specific alterations of the proteins codified by these two genes.

## Conclusions

The clinical and pathological characterization of multiple NF lesions may be used as screening for the identification of families at risk of NF1. Our data indicate that a proclivity to multiple tumors arising in the same subject, and a higher tumor burden per family are the most relevant findings observed in affected patients with the NF1 mutation. Overall, the current findings indicate a possible strategy that may be followed for the identification and management of NF1 patients for which the early diagnosis of abdominal manifestations is crucial because of the risk of malignancy, organic complications or hemorrhagic-obstructive complications such as in the case of tumors of the gastrointestinal tract.

## Consent

Written informed consent was obtained from the patients for publication and any accompanying images.

## Authors' contributions

GP: Study concept and design; acquisition of data; drafting of the manuscript. LL: Contribution to drafting of the manuscript; immunohistochemical analysis and interpretation of data. MP: Genetic analysis and interpretation of data. EB: Acquisition of data. DM: Biomolecular and genetic analysis and interpretation of data; AP: Acquisition and interpretation of data. SS: Study supervision. All authors read and approved the final manuscript.

## Competing interests

The authors declare that they have no competing interests.
